# A new superfluity deep learning model for detecting knee osteoporosis and osteopenia in X-ray images

**DOI:** 10.1038/s41598-024-75549-0

**Published:** 2024-10-25

**Authors:** Soaad M. Naguib, Mohammed K. Saleh, Hanaa M. Hamza, Khalid M. Hosny, Mohamed A. Kassem

**Affiliations:** 1 Information Systems Department, Zagazig University, Zagazig, 44519 Egypt; 2 Department of Orthopedic Surgery, Zagazig University, Zagazig, 44519 Egypt; 3 Information Technology Department, Zagazig University, Zagazig, 44519 Egypt; 4 Dept. of Robotics and Intelligent Machines, Kafr El Sheikh University, Kafr El Sheikh, Egypt

**Keywords:** Knee, X-ray images, Osteoporosis/osteopenia, Deep learning, Superfluity, Health care, Medical research

## Abstract

This study proposes a new deep-learning approach incorporating a superfluity mechanism to categorize knee X-ray images into osteoporosis, osteopenia, and normal classes. The superfluity mechanism suggests the use of two distinct types of blocks. The rationale is that, unlike a conventional serially stacked layer, the superfluity concept involves concatenating multiple layers, enabling features to flow into two branches rather than a single branch. Two knee datasets have been utilized for training, validating, and testing the proposed model. We use transfer learning with two pre-trained models, AlexNet and ResNet50, comparing the results with those of the proposed model. The results indicate that the performance of the pre-trained models, namely AlexNet and ResNet50, was inferior to that of the proposed Superfluity DL architecture. The Superfluity DL model demonstrated the highest accuracy (85.42% for dataset1 and 79.39% for dataset2) among all the pre-trained models.

## Introduction

Osteoporosis is a disease that causes a reduction in bone mass and bone architecture disruption, increasing the risk of fracture and compromised bone strength^1^. Osteopenia describes a drop in body mass density (BMD) below normal levels but not low enough to meet the diagnostic threshold for osteoporosis. Osteoporosis is a major public health problem and one of the most common bone diseases^2^. Women and older people are more likely to experience it. Even in younger people, osteoporosis can occur because of specific medical disorders.

Currently, over 200 million people all over the world have osteoporosis^3,37^. It is predicted that by 2025, the global fracture rate may rise by 50%^4^. Osteoporosis is considered a silent disease, as there are commonly no symptoms until the first fracture occurs^3^. Bone deformity, osteoarthritis, and fractures are the most serious complications of osteoporosis^5–8^ since osteoporosis is a disease that can affect all parts of the skeleton^9^.

To detect osteoporosis, a bone density scan should be taken. For identifying BMD, DEXA is the standard test for identifying osteoporosis^10^. However, the DُEXA scans have some drawbacks, such as not being widely available, longer scan times, and the skilled personnel needed to perform the scan. In addition, DEXA devices are expensive, which makes these devices unsuitable for general screening in primary healthcare^10–12^. Therefore, it would be useful to reduce time and expenses by detecting osteoporosis/osteopenia from digital X-ray images. Few studies have been done to measure bone density directly by analyzing X-ray images^4^. X-ray imaging is the most common and cheap modality used to scan bones. Therefore, the early detection of osteoporosis/ osteopenia with widely used X-ray images would be useful and cost-effective in preventing fractures and other bone complexities. However, X-rays only help in qualitative assessment (only detecting osteoporosis or osteopenia) and cannot be considered a quantitative assessment tool like DEXA^13^. The recent advancement of artificial intelligence (AI) for automated image interpretation has led researchers to analyze images using deep learning (DL) to detect diseases and classify images. Many researchers have conducted studies to detect osteoporosis. For example, Sukegawa et al.^11^ used CNN models to detect osteoporosis from dental panoramic radiographs. The results indicated that CNN can accurately detect osteoporosis from dental radiographs. In addition, Jang et al.^14^ used DL (VGG16) to predict osteoporosis from hip radiographs. The result shows an accuracy of 81.2%, sensitivity of 91.1%, and specificity of 68.9%. The study concluded that DL models are useful screening tools when predicting osteoporosis in clinical settings. Li et al.^15^ used machine learning and computed tomography (CT) attenuation to predict osteopenia and osteoporosis for all bones visible on chest CT scans. Results showed that CT attenuation and machine learning are acceptable for predicting BMD and osteopenia/osteoporosis. Mane et al. 2023^16^ used CNN models to detect and classify osteoporosis on human spine X-ray images. The VGG16 with Random Forest achieved the highest accuracy with 95%. Respecting osteoporosis detection from knee X-rays, Wani and Arora^15^ used CNN to classify knee X-ray images into normal, osteopenia, and osteoporosis classes. They utilized four well-known models: AlexNet, vgg19, vgg16, and ResNet. The best accuracy was 78.95% achieved by Alexnet. Kumar et al.^17^ proposed a deep-learning model to classify Osteoporosis using knee X-ray images. They achieved an accuracy rate of 82.61%. Xie et al.^18^ finetuned a deep convolutional neural network (ImageNet) on knee X-rays to diagnose osteoporosis. They achieved an accuracy of 0.728 and a sensitivity of 0.774.

Detecting the disease as early as possible is crucial to prevent further bone density loss and complications related to knee osteoporosis and osteopenia. However, the traditional methods for detecting bone-related conditions are subjective and subject to human error. In contrast, automated and accurate diagnosis through deep learning models can enhance reliability. Medical image datasets often exhibit patient demographics, imaging equipment, and condition variations. So, robust models are needed to handle such diversities effectively. In addition, minimizing false positives and negatives is critical in medical diagnosis to avoid unnecessary treatments or overlook potential health risks. By automating the detection process through deep learning models, healthcare professionals can focus on treatment planning and patient care, reducing their time on medical investigation tasks. This study provides the following contributions.

1. We proposed two superfluity mechanisms to build a novel DL model for detecting knee osteoporosis/osteopenia.
2. This model has the potential to improve clinical efficiency by providing a reliable, automated tool for initial assessment as well as reducing false positives and false negatives, thereby enhancing the precision with which knee osteoporosis and osteopenia can be detected and allowing healthcare providers to prioritize patient care more effectively and allocate their time more effectively.

## Methods

DL-based CNN techniques have grown in popularity recently among computer-aided diagnosis (CAD) systems for medical image analysis^19^ because of their cutting-edge results in detecting a variety of diseases from images, such as breast cancer detection^20^, cervical spine fracture and dislocation^21^, skin cancer detection^22^, early diagnosis of Alzheimer’s^23^, brain tumor classification^24^, classifying uni-bicompartmental knee^25^, Pelvis Fracture Detection^26^, human activity recognition^27^, etc. Regarding classifying medical images, CNNs like AlexNet, ResNet-50, and GoogleNet^28–30^ have produced cutting-edge findings. The primary problem with using CNN classifiers is that enormous amounts of labeled data for training are needed. However, finding such a large dataset in the medical field is very challenging. So, transfer learning has been utilized to overcome these challenges^31^.

Numerous CAD systems, including DL, are suggested for osteoporosis diagnosis at various bone sites, such as the hand, hip, spine, and tooth. Still, research is lacking to identify knee osteoporosis.

It has been estimated that half of patients older than 50 may suffer from knee fractures. A high 1-year mortality rate of 22% is observed in elderly patients who suffer femoral fractures, with less function and poor quality of life. An early detection method is needed to identify the presence of osteoporosis in the knee bone, prevent fractures, and decrease treatment costs^32^. The following subsection provides a brief overview of the AlexNet and Resnet-50 models. AlexNet holds historical significance as one of the pioneering models that demonstrated the potential of deep learning in image classification. Its success in the ImageNet Large Scale Visual Recognition Challenge (ILSVRC) 2012 makes it a benchmark architecture for this specific task.

On the other hand, ResNet-50 introduces the concept of residual learning, addressing challenges associated with training very deep networks. Including skip connections allows for the construction of deeper architectures, which can capture more intricate features in the data. We have opted for AlexNet and ResNet-50 for specific reasons. The other CNN architectures can be computationally expensive and introduce additional complexities that may not be necessary for our particular task.

### AlexNet

**Fig. 1:**
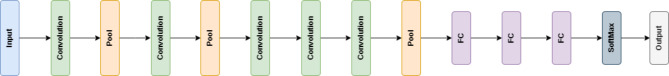
Alexnet architecture.

AlexNet is a pioneering convolutional neural network (CNN) architecture that significantly contributed to advanced deep learning in computer vision. Alexnet is a well-known deep-learning model proposed by Krizhevsky et al.^28^. It achieved high accuracy at the ImageNet LSVRC-2010 classification of 1.2 million HR (high resolution) images containing 1000 different classes with a top error rate of 15.3%. It fared better than the most recent architectures. The network comprises three fully connected layers, three max-pooling layers, five convolutional neural networks, and a softmax classifier. Figure 1 shows the full architecture of Alexnet.

### ResNet-50

He et al.^29^ proposed the ResNet CNN architecture. Resnet won the 2015 ILSVC competition, lowering the error rate to 3.6%. It has 152 layers, making it a very deep network. ResNets are constructed from many residual blocks. By utilizing skip connections, residual blocks assist in feeding the activation of one layer to a deeper layer in the network, facilitating quicker system training.

According to the number of network layers, ResNet comes in various forms, including ResNet-18, ResNet-34, ResNet-50, ResNet-101, and ResNet-152. Due to the size of our dataset in this study, we used the ResNet-50 architecture.

## The proposed algorithm

We proposed two models to classify the knee into normal, osteopenia, or osteoporosis. First, we develop a novel deep-learning model using a superfluity mechanism. Second, we use transfer learning to predefine the DL architectures Alexnet and Resnet-50. The second model using Alexnet, and Resnet-50 is used to compare the obtained results from the proposed deep learning with the superfluity mechanism.

### Superfluity deep learning model

CNN is a type of deep neural network. The intermediate levels of the CNN are built on the convolutional principle. The mathematical operation known as convolution involves modifying one function with another to produce a new function with some changed characteristics. When processing images, CNNs are used to reduce the size of the images without sacrificing their fundamental information by convolving the image with a filter that is shorter in length and width to extract patches automatically from the image.

CNN’s strength comes from its ability to learn the image data straight from the image without additional feature extraction or object segmentation techniques in other machine learning techniques. Numerous CNNs have been created to address different issue types, and while they differ in a few key ways, their fundamental elements remain the same. The convolutional, pooling, and fully connected layers are the three different kinds of layers that make up CNNs. The convolutional layer oversees the use of various filters to acquire the feature representations of the input images which extract the patches automatically from the image. With the representations being downscaled, the pooling layer lowers the computations and parameters to accomplish shift-invariance. Typically, it is positioned between the two convolutional layers. The network could contain any number of convolutional and pooling layers. We can obtain them by correctly stacking the feature maps holding the higher-level representations. One or more fully connected (FC) layers are present after the stacked convolutional and pooling layers and before the output layer to perform the reasoning job.

The output from one layer of a deep neural network is fed into the subsequent layer, which is arranged serially. It is challenging to raise and increase the depth levels. More images are needed for training deeper networks because there are many parameters that these networks contain to generalize the network. So, going deeper is not the best solution, especially with medical datasets containing few images. In addition, going deeper increases the degradation problem and overfitting.

To solve certain problems that arise in the course of training very deep neural networks. Superfluity learning seeks to adequately remediate the issues of inadequate datasets, vanishing gradients, and degradation in extremely deep networks to improve on the depth neural networks proposed. The main point is to apply branches that add one or several layers to the forward pass and the backpropagation step.

**Fig. 2:**
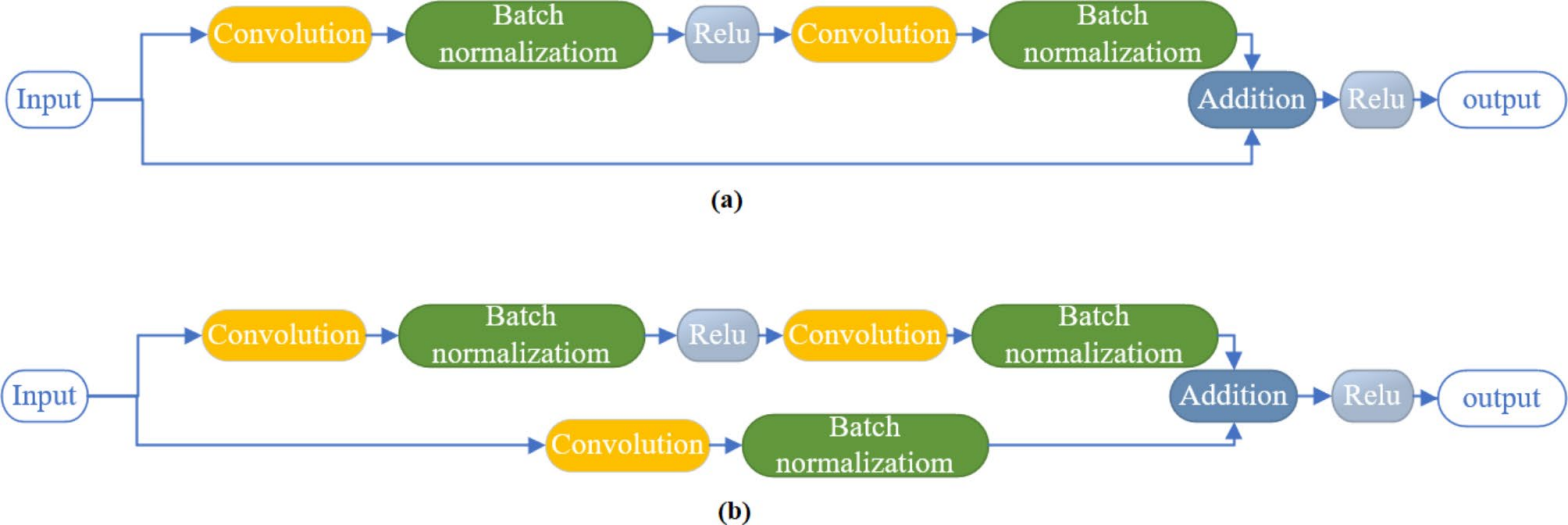
Superfluity mechanism, (a) superfluity mechanism 1 (SM1), and (b) superfluity mechanism 2 (SM2).

The suggested model employs superfluity learning to get around image degradation and scarcity. Superfluity learning enhances information flow and reformulates layers by surpassing layer input connections, overcoming degradation. Instead of passing the previously extracted features through a single path, the superfluity learning mechanism passes the extracted features from the previous layers through two different paths. Then, the extracted features from the two paths are concatenated together. A superfluity block consists of two or more convolutional layers with a bypass of them. The bypass connection adds the input of the block directly to its output. We proposed two superfluity blocks. As shown in Fig. 2(a), the first superfluity block consists of two convolution layers each layer followed by a batch normalization layer, and the activation function was a non-linear rectified linear activation function (ReLU). Then the input is directly passed by the bypass and fused with its output. The second superfluity block is shown in Fig. 2(b). the construction of the second block is the same as the first block but the bypass connection contains a convolutional layer and batch normalization layer. Then, the input of the feature map is passed through the stacked layer and the bypass connection, and then the output is fused to construct one feature map. These blocks are combined to form the suggested deep superfluity network. The superfluity blocks extract the image patches automatically by maintaining different filter sizes in the same block. The different filter sizes construct the superfluity mechanism to extract high and low features that differentiate between knee diagnoses.

**Fig. 3:**
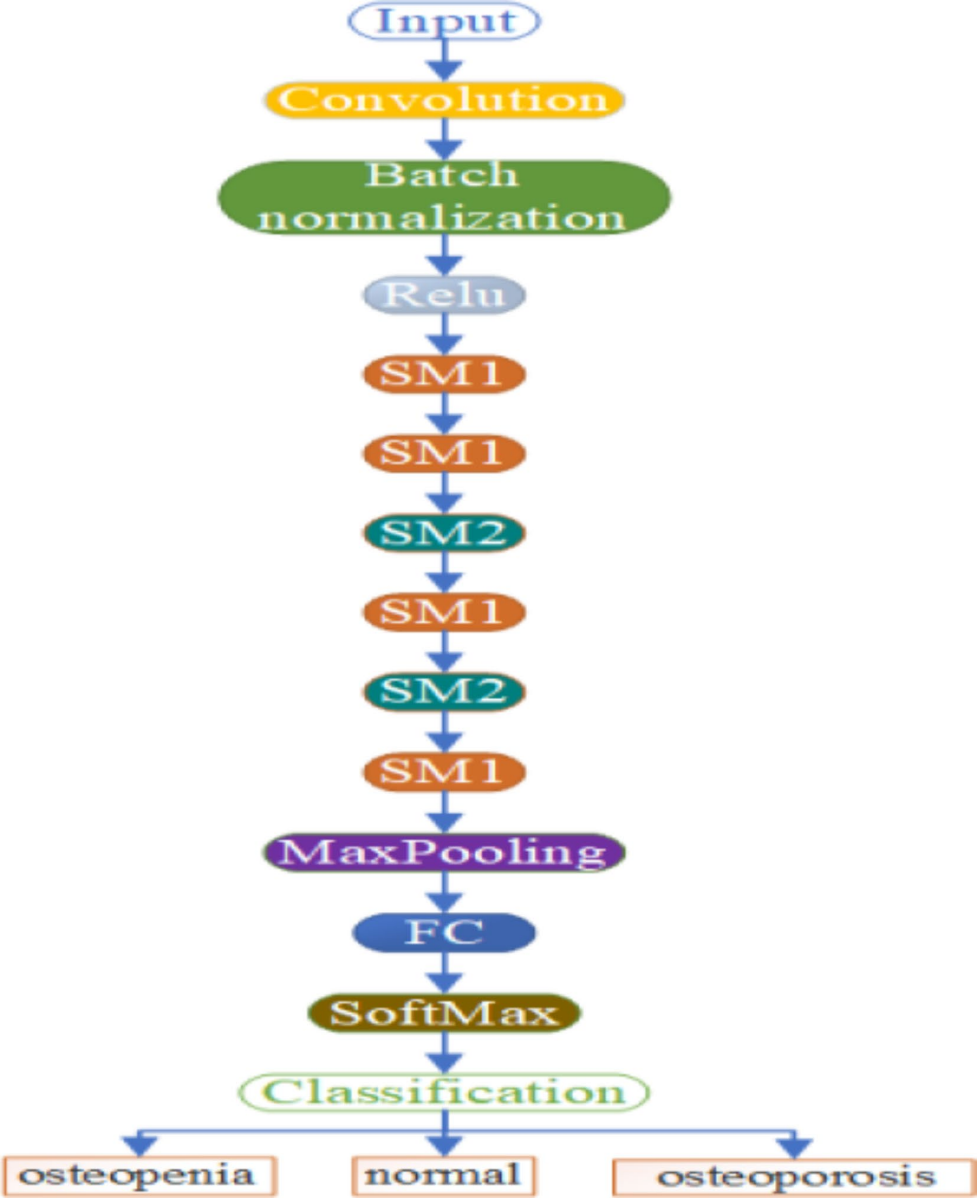
The full architecture of the DL model where SM1 is the superfluity mechanism 1, SM2 is the superfluity mechanism 2, and FC is fully connected.

The proposed DL model consists of 54 layers. The first layer is an input layer with a width and height of 300 × 300. Then, the input layer is followed by different layers and superfluity mechanisms. These layers are convolution, batch normalization, element-wise addition layer of two inputs, MaxPooling, fully connected, SoftMax, and classification layer. The non-linear rectified linear activation function (ReLU) is used to solve the issue of vanishing gradients. Figure 3 shows the full architecture of the proposed method.

Superfluity enhances the model’s robustness. It helps the model perform well even when certain components or features are compromised or noisy. The additional pathways or connections in a superfluity model contribute to improved generalization, allowing the model to adapt better to diverse datasets and variations in input conditions. Superfluity mechanisms can mitigate the risk of overfitting, which is particularly beneficial when dealing with limited or imbalanced datasets. Superfluity can make the training process more stable and accelerate convergence. The presence of redundant pathways may enable the model to learn more efficiently, especially for complex tasks.

### Transfer learning

In machine learning, reusing a model already trained on a different task or problem is known as transfer learning. This approach enables a computer to apply knowledge from one task to enhance its generalization capabilities for another task. Due to its ability to train deep neural networks with little data, it is now preferred in DL.

In transfer learning, the pre-trained model is divided into feature extraction and classification layers. The feature extraction layers extract the features from the source task, while the classification layers are task-specific. Getting the millions of annotated images needed to train a CNN is extremely difficult in the medical industry. So, the feature extraction layers are frozen during fine-tuning to retain their learned knowledge. Here, we propose to replace the last layer responsible for the classification (two layers) with a new fully connected layer and classification layer. The advantages are saving training time, improved neural network performance (generally), and fewer data requirements.

Mathematically, transfer learning involves fine-tuning the parameters of a pre-trained model $\:M$on a source task. $\:{T}_{s}$ to improve its performance on a target task $\:{T}_{t}$The adapted model is denoted as $\:{M}^{{\prime\:}}$, and the fine-tuning process can be expressed as:

$$\:{M}^{{\prime\:}}=finetune\:(M,{T}_{t})$$

## Experimental results

The main goal of this study is to build a system for classifying three forms of the knee from X-ray images. As discussed previously, the proposed model is novel. So, different testing and measures must be performed to ensure the proposed method’s reliability. MATLAB 2022a 64-bit was used to develop the deep convolutional models. On a computer equipped with 16 GB of RAM, a Graphics Processing Unit (GPU), and an Intel (R) Core (TM) Processor i7-8550U @ 1.80 GHz, all of the experiments were carried out under Windows. The hyperparameters for the training options for the proposed and the pre-trained model are summarized in Table 1.

**Table 1:** Training hyperparameter values.

Name	Value
Initial Learn Rate	0.001
Max Epochs	30
Batch Size	10
Shuffle	Every epoch
Learning drop factor	After 4 epochs if the accuracy has not increased
Validated	Every 50 iteration
Optimizer	Stochastic Gradient Descent With Momentum (SGDM)

The proposed model was trained, validated, and tested on two datasets. This section will describe the two datasets by clarifying their references and classifying them into three groups. After that, the results of the experiments will be presented later in the next sections. In addition, we would like to clarify that the datasets were obtained online but not used directly; they were reviewed and audited by specialized orthopedic surgeons.

**Fig. 4:**
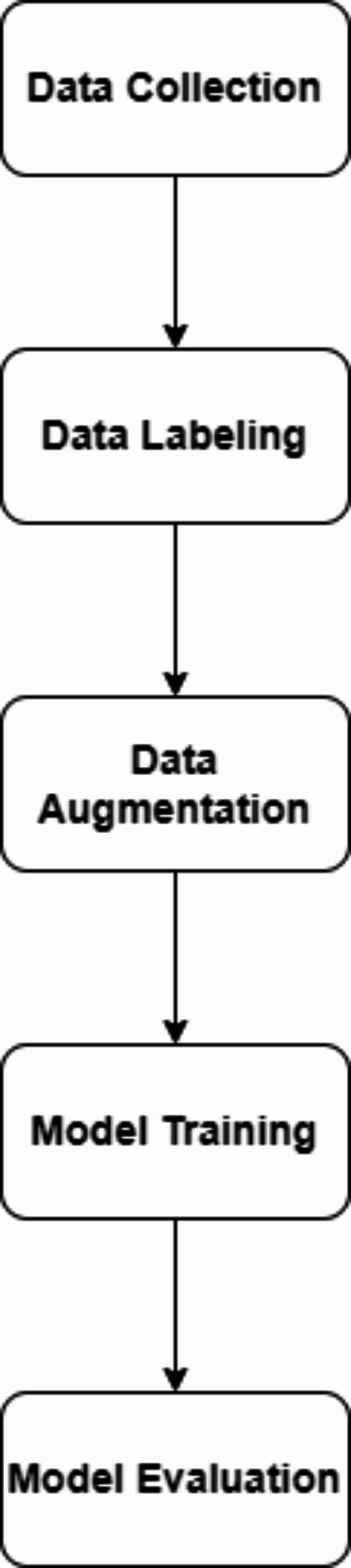
The overall process of the proposed method.

**Fig. 5:**
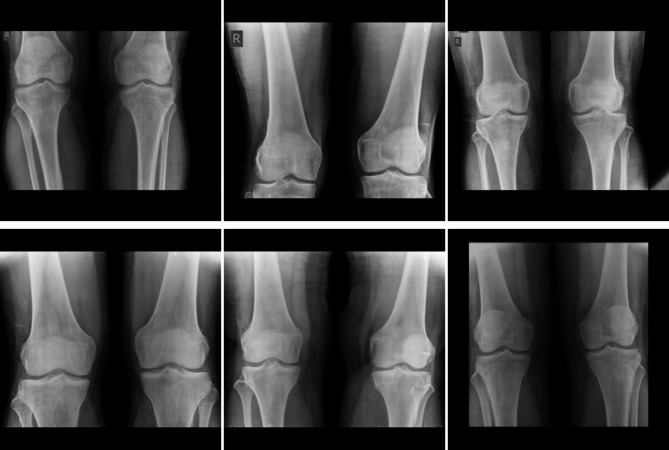
Samples from the first dataset (normal images).

**Fig. 6:**
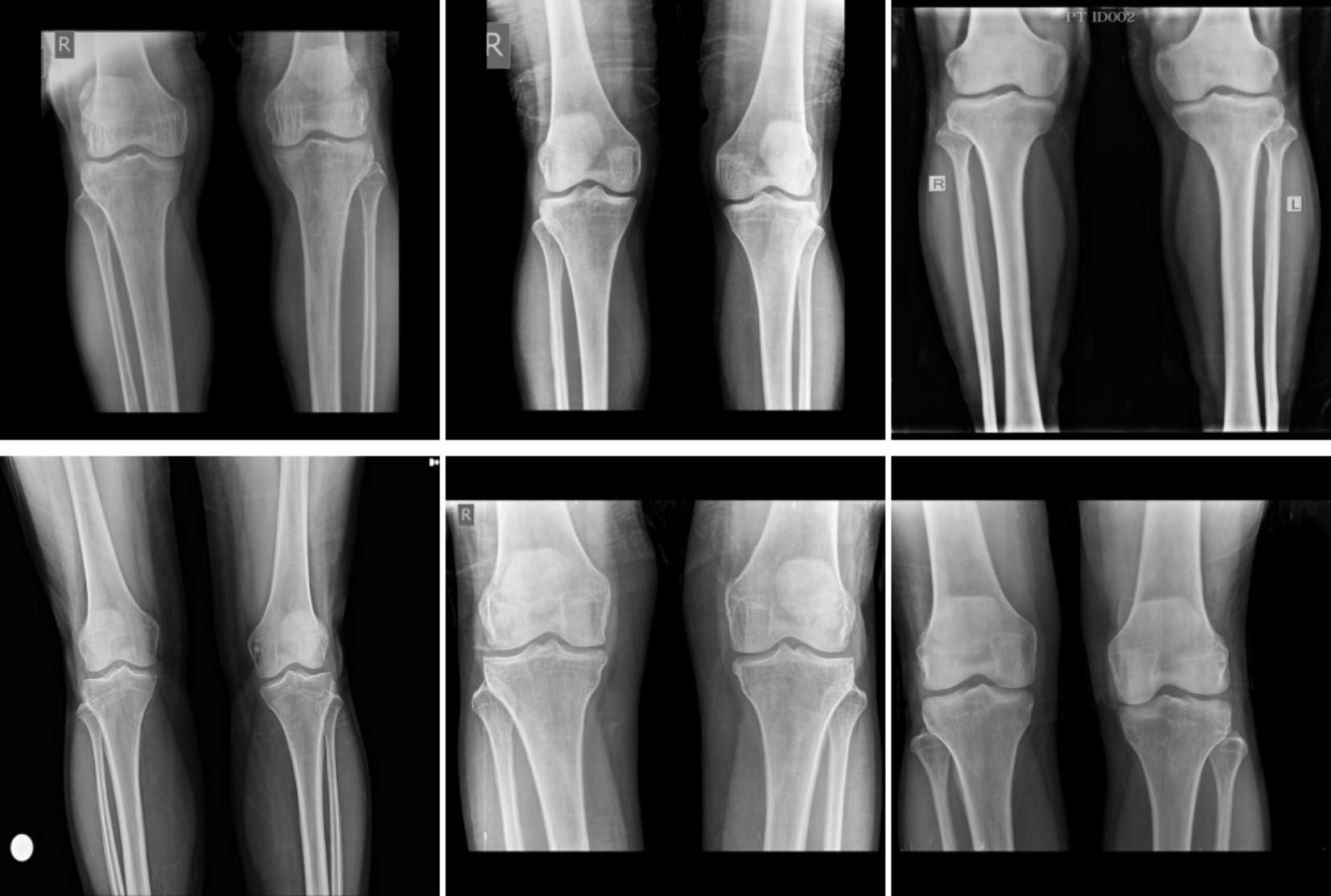
Samples from the first dataset (osteopenia images).

**Fig. 7:**
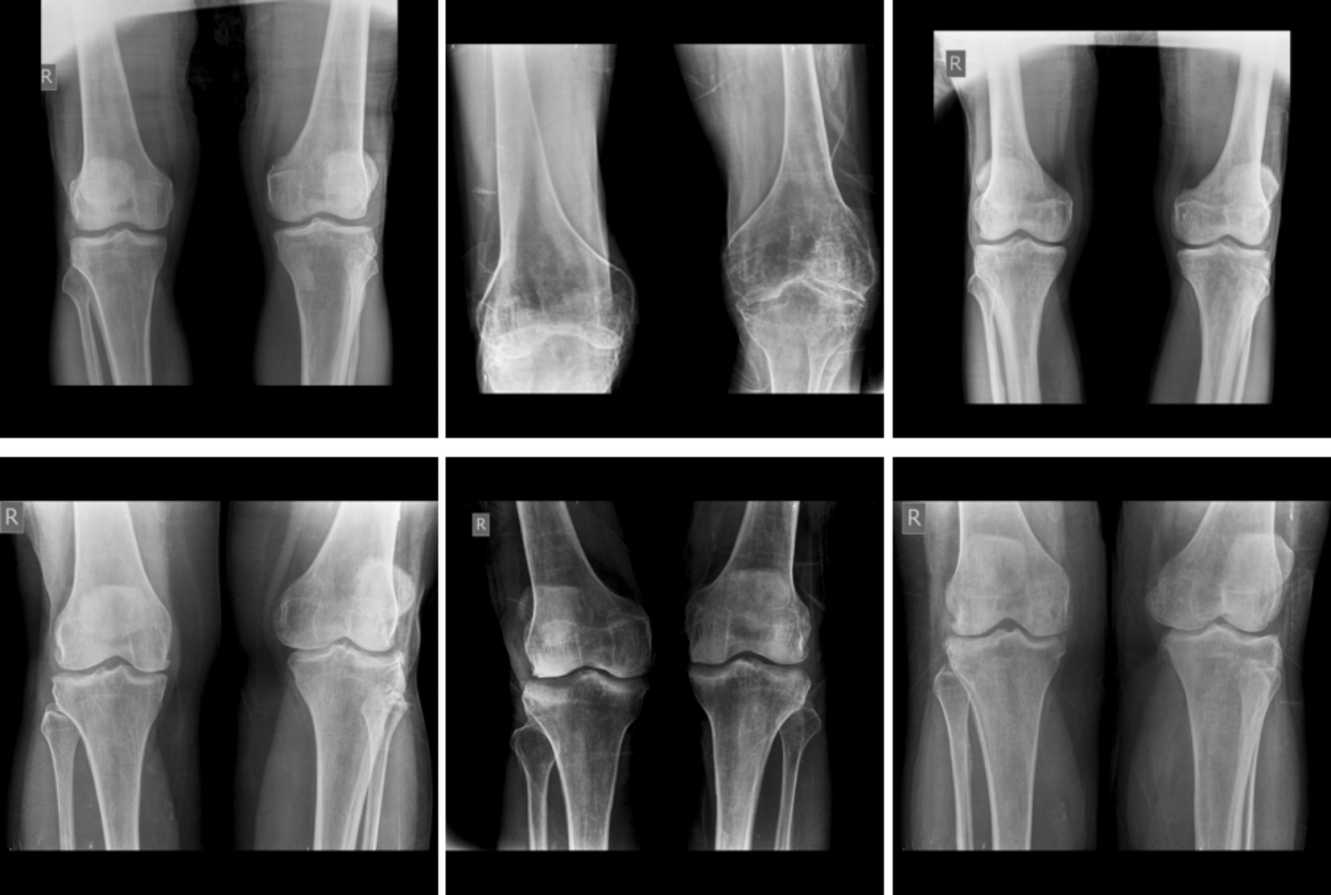
Samples from the first dataset (osteoporosis images).

The first dataset was obtained from Mendeley Data^34^. It has been collected in the Unani and Panchkarma Hospital, India, from 21-12-2019 to 31-12-2019. It consists of 240 X-ray images. The images are classified into three classes. The overall process is shown in Fig. 4. The first class includes 80 normal images, as shown in Fig. 5. Figure 6 shows the second class includes 129 osteopenia images. Finally, the third class includes 30 osteoporosis cases, as shown in Fig. 7.

**Fig. 8:**
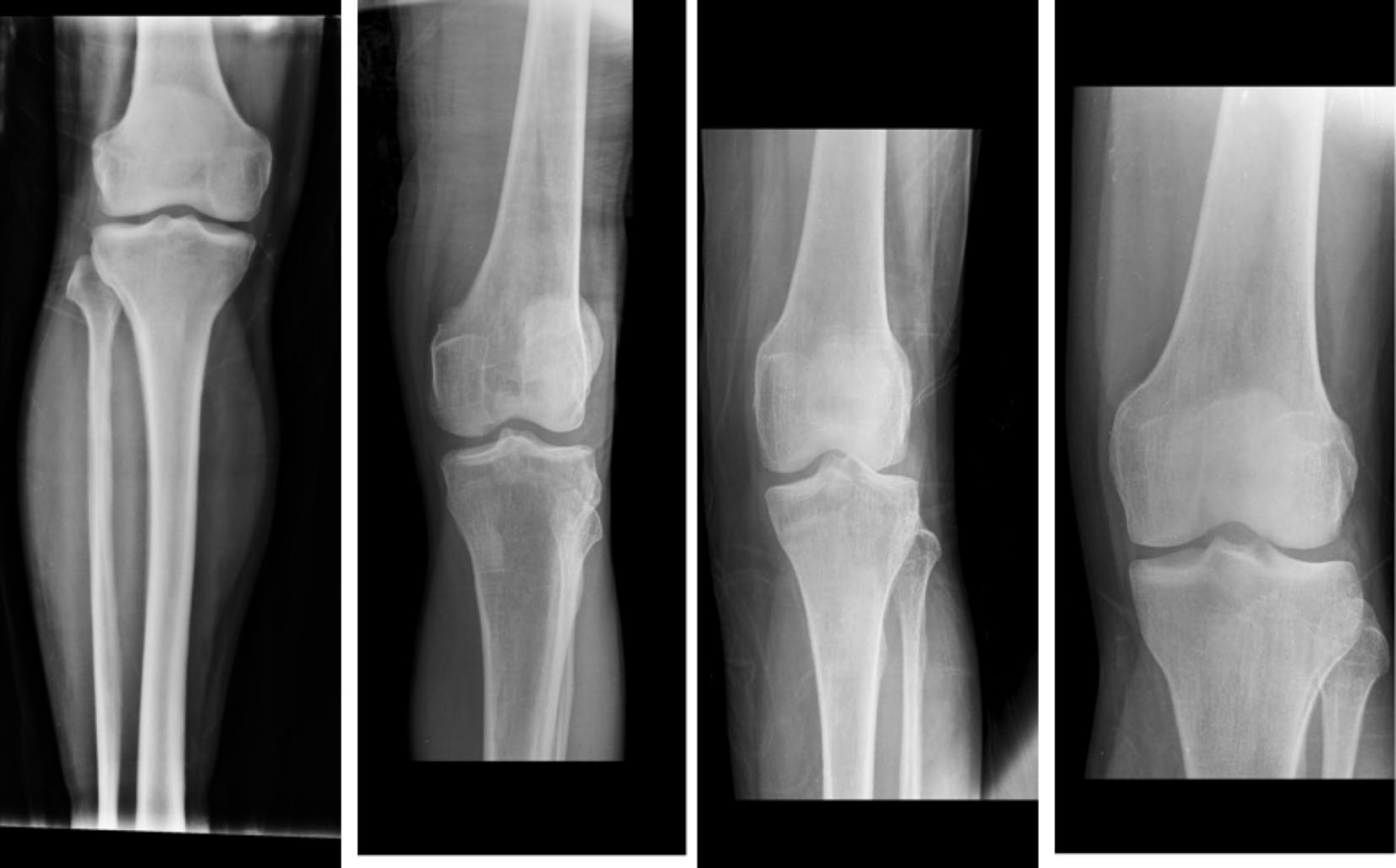
Samples from the second dataset (normal images).

**Fig. 9:**
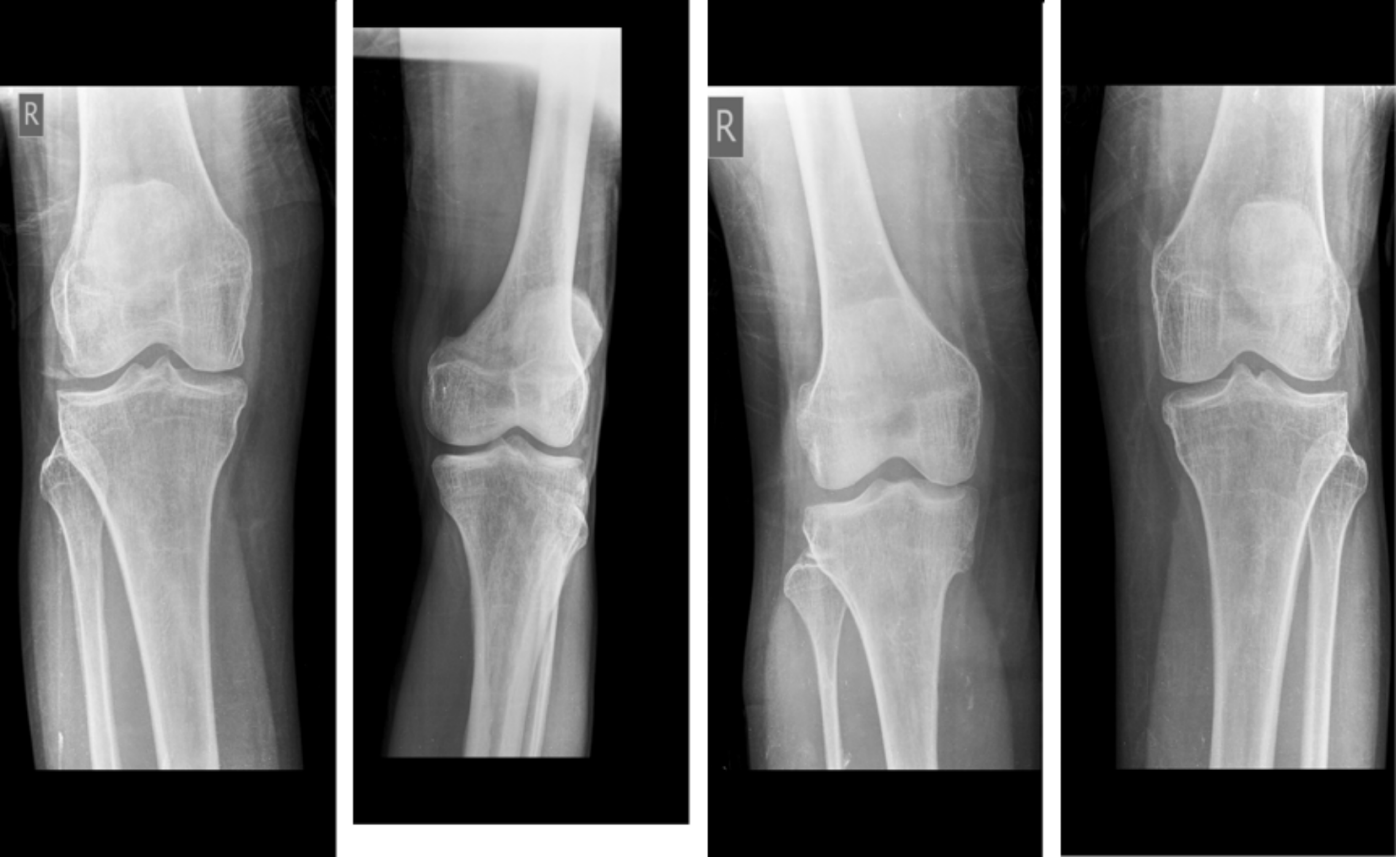
Samples from the second dataset (osteopenia images).

**Fig. 10:**
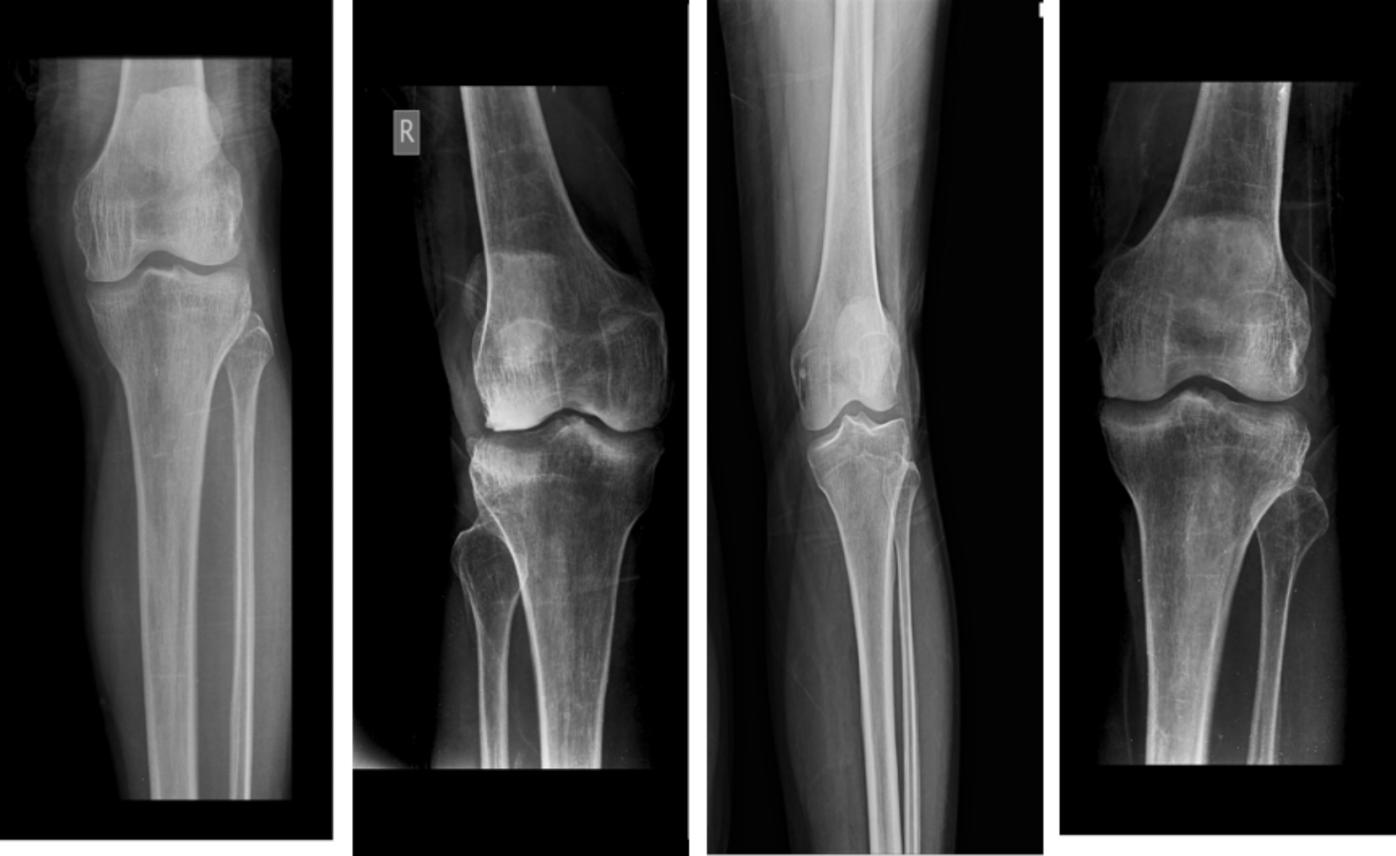
Samples from the second dataset (osteoporosis images).

The second dataset was obtained from Kaggle Datasets^35^. It consists of 371 X-ray images. The first class includes 159 normal images shown in Fig. 8. The second class includes 153 osteopenia images shown in Fig. 9. Finally, the osteoporosis class includes 59 images shown in Fig. 10. These images were annotated and validated by orthopedic surgery specialists who classified the images as normal, osteopenia, or osteoporosis.

### Performance metrics

At a ratio of approximately 70:10:20, each image from the primary dataset was randomly assigned to the training, validation, and testing sets. The test dataset was used to provide actual predicted results. The validation dataset was used to select hyperparameters and stopping conditions, and the training set was used to train the model. The model was used to distill low-level and high-level features from the extracted patches automatically. Ultimately, the model was structured to classify normal, osteoporosis, and osteopenia cases.

Both continuous variables and descriptive statistics were expressed as percentages. We used the ROC curve to classify osteoporosis, osteopenia, and normal cases for diagnosis. Our study presented the confusion matrix as a 3 × 3 contingency table listing the number of true positives. $\:{(t}_{p})$, false negatives $\:\left({f}_{n}\right)$, false positives ($\:{f}_{p})\:$and true negatives ($\:{t}_{n})$. The training parameters are fixed for all experiments. These parameters were 0.001 for the initial learning rate, the learn drop factor was 4, momentum was 0.95, the minibatch size equals 10, the maximum number of iterations was 30, and shuffle the images every iteration. These measures are computed from the following equation^34^:

1 $$\:\text{Accuracy}\:=\:\frac{{\text{t}}_{\text{p}}+{\text{t}}_{\text{n}}}{{\text{t}}_{\text{p}}+{\text{f}}_{\text{p}}+{\text{f}}_{\text{n}}+{\text{t}}_{\text{n}}}$$

2 $$\:\text{Sensing}\:=\:\frac{{\text{t}}_{\text{p}}}{{\text{t}}_{\text{p}}+{\text{f}}_{\text{n}}}$$

3 $$\:\text{Specificity}\:=\:\frac{{\text{t}}_{\text{n}}}{{\text{f}}_{\text{p}}+{\text{t}}_{\text{n}}}$$

4 $$\:\text{Precision}\:=\:\frac{{\text{t}}_{\text{p}}}{{\text{t}}_{\text{p}}+{\text{f}}_{\text{p}}}$$

5 $$\:\text{F}-\text{score}\:=\:\frac{{\text{t}}_{\text{p}}}{{\text{t}}_{\text{p}}+\frac{1}{2}\:{(\text{f}}_{\text{p}}\:+\:{\text{f}}_{\text{n}})}$$

### Results and discussions

**Fig. 11:**
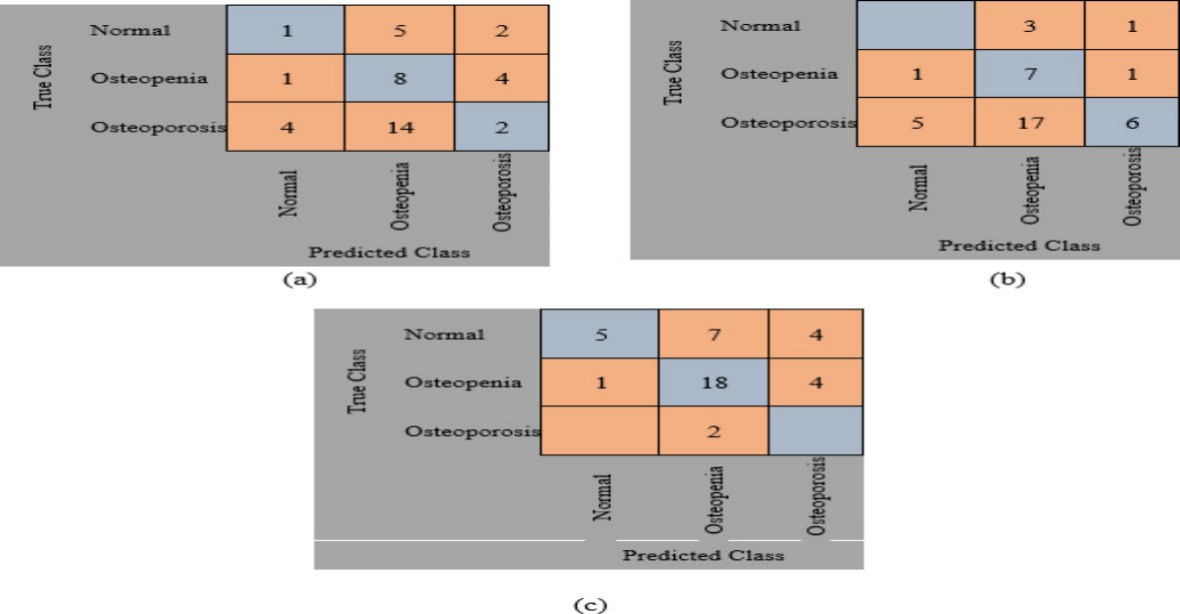
Confusion matrix for the proposed method for dataset 1 without augmentation where (a) Confusion matrix of Alexnet, (b) Confusion matrix of resnet50, (c) Confusion, matrix of proposed superfluity DL.

**Fig. 12:**
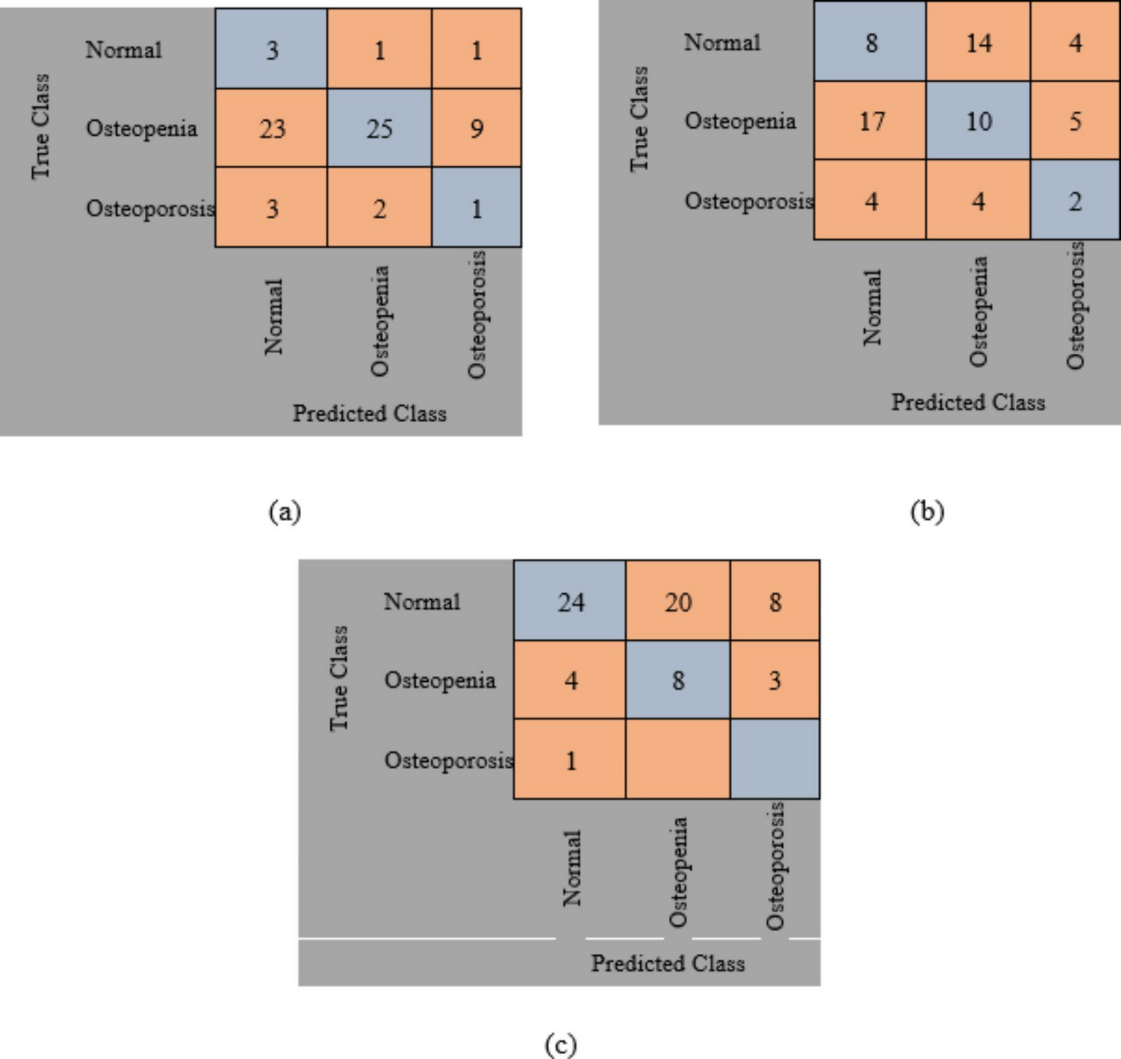
Confusion matrix for the proposed method for dataset 2 without augmentation where (a) confusion matrix of Alexnet, (b) Confusion matrix of resnet50, (c) Confusion matrix of the proposed superfluity DL.

**Fig. 13:**
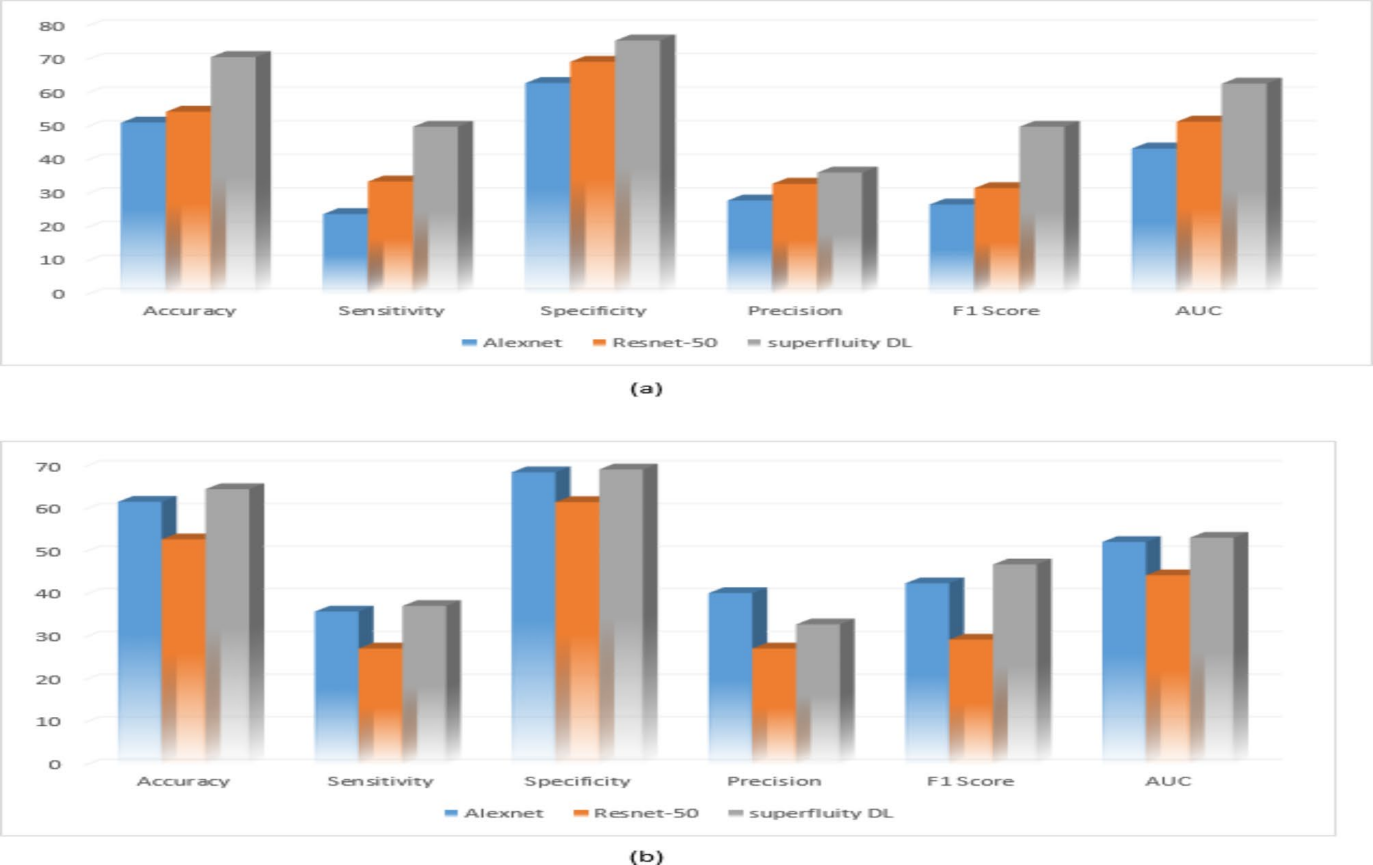
(a) Dataset 1 comparison before augmentation, (b) Dataset 2 comparison before augmentation.

**Fig. 14:**
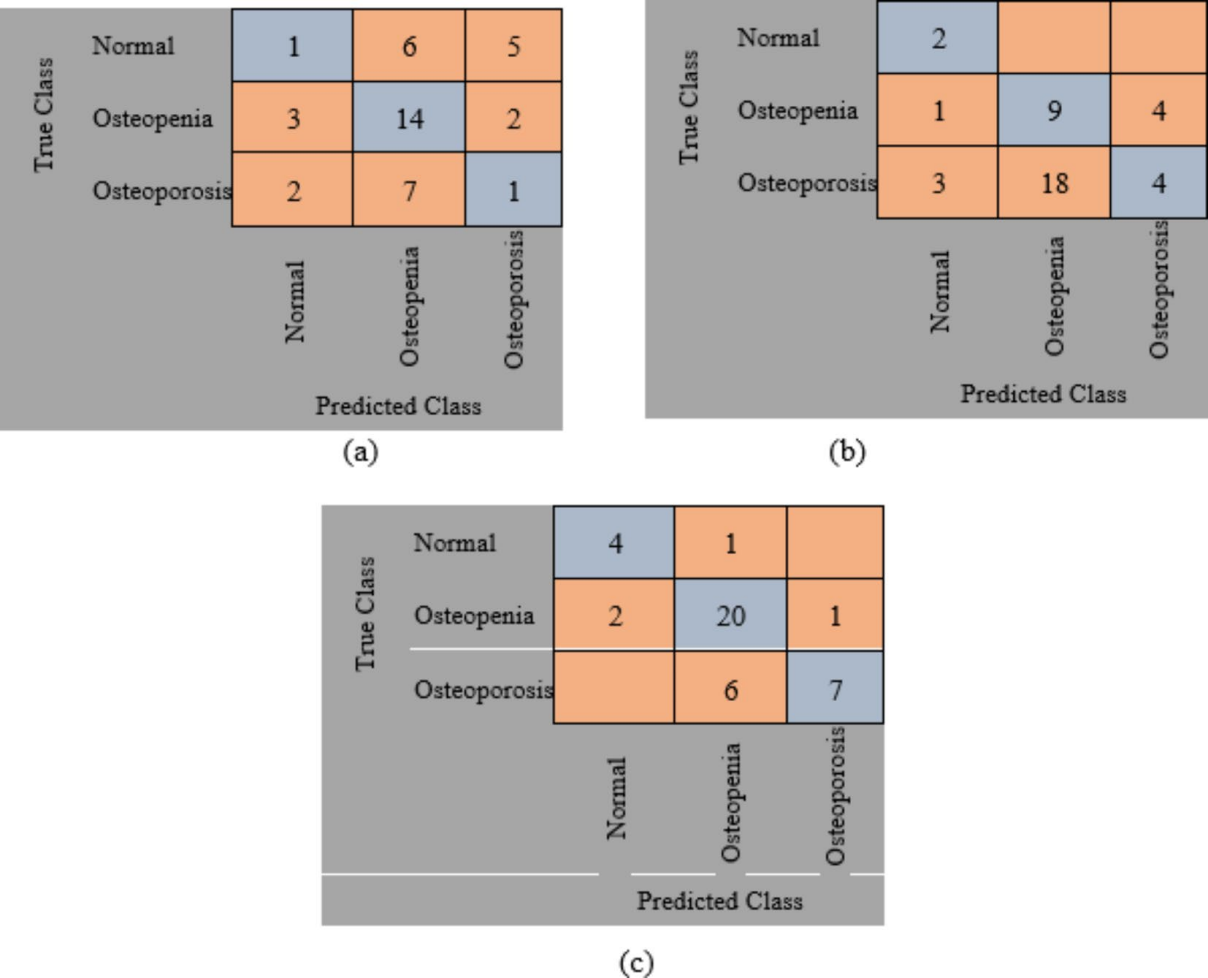
Confusion matrix for the proposed method for dataset 1 after augmentation where (a) confusion matrix of Alexnet, (b) Confusion matrix of resnet50, (c) Confusion matrix of the proposed superfluity DL.

**Fig. 15:**
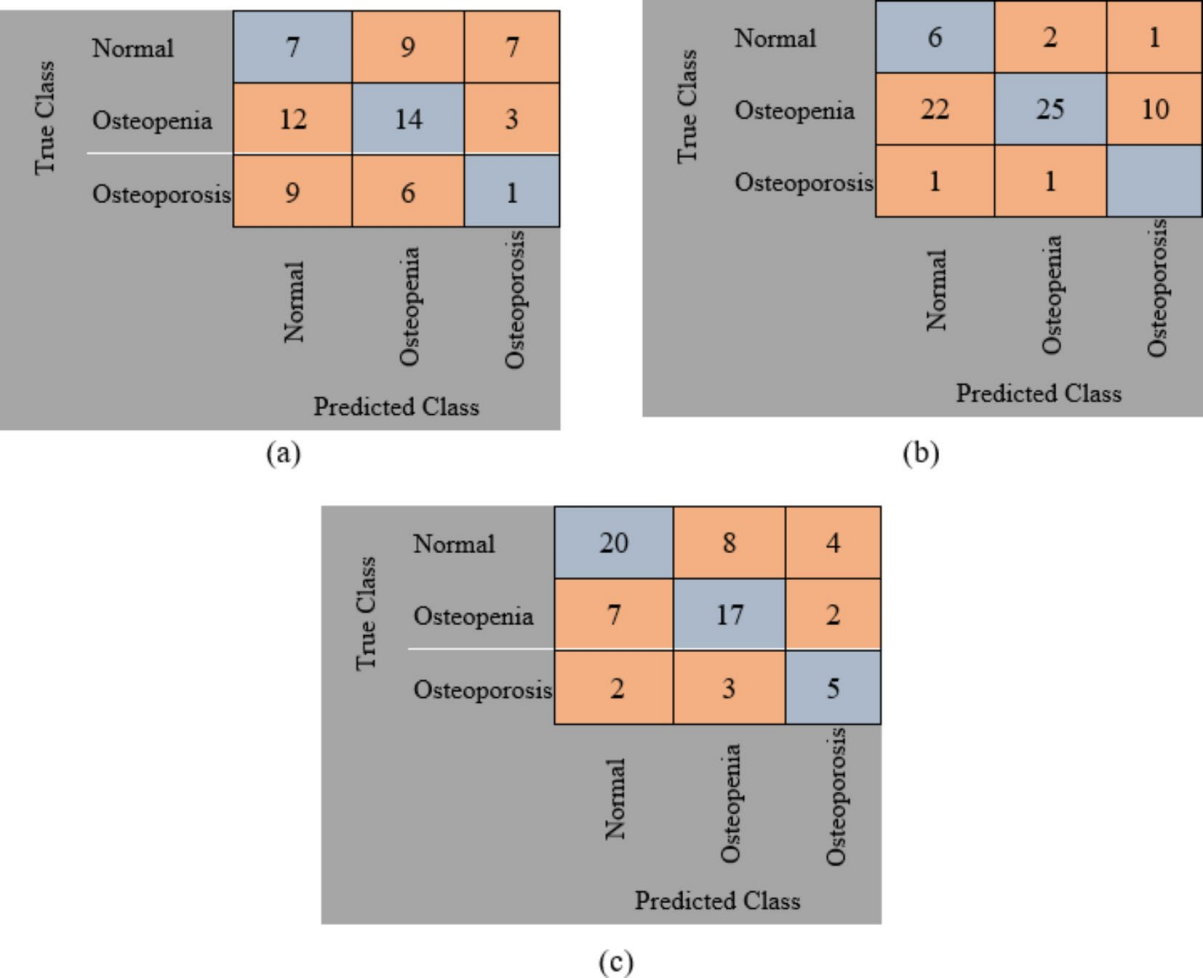
Confusion matrix for the proposed method for dataset 2 after augmentation where (a) confusion matrix of Alexnet, (b) Confusion matrix of resnet50, (c) Confusion matrix of the proposed superfluity DL.

The original datasets’ images have been used separately for training, validating, and testing the proposed DL model. The confusion matrix of the proposed models is shown in Figs. 11 and 12. Figure 13 shows the performance comparison between two data sets, and the obtained measures are listed and depicted in Table 2 for Dataset 1 and Table 3 for Dataset 2. Next, the images were augmented using different parameters such as Gaussian noise, rotation= [-45 45], x-translation= [-50 50], y-translation= [-50 50], and x-reflection, y-reflection in addition to Gaussian noise^36^. The number of images after augmentation was 400, 696, and 150 for normal, osteopenia, and osteoporosis for the first dataset. At the same time, the number of images after augmentation in the second dataset becomes 551, 557, and 219 for normal, osteopenia, and osteoporosis, respectively. The confusion matrix of the augmented images trained by the proposed model is shown in Figs. 14 and 15. At the same time, the obtained measures are listed and depicted in Tables 4 and 5 for Datasets 1 and 2.

**Table 2:** Performance measures for dataset 1 without augmentation.

	Accuracy (%)	Sensitivity (%)	Specificity (%)	Precision (%)	F1 Score (%)	AUC (%)
Alexnet	51.22	24	63	28	26.83	43.5
Resnet-50	54.47	33.67	69.33	33	31.71	51.5
Superfluity DL	**70.73**	**50**	**75.67**	**36.33**	**50**	**62.84**

**Table 3:** Performance measures for dataset 2 without augmentation.

	Accuracy (%)	Sensitivity (%)	Specificity (%)	Precision (%)	F1 Score (%)	AUC (%)
Alexnet	61.76	36	68.67	**40.33**	42.64	52.33
Resnet-50	52.94	27.33	61.67	27.33	29.41	44.5
Superfluity DL	**64.71**	**37.33**	**69.33**	33	**47.06**	**53.33**

From the previous confusion matrices, the pre-trained models, Alexnet and Resnet50, were lower than the proposed Superfluity DL architecture. In the obtained results, Alexnet obtained higher measures than Resnet50. At the same time, the proposed Superfluity DL was the best compared to the other pre-trained models. Still, the obtained results need to be enhanced, so we carried out augmentation to the dataset. The dataset is very small. In addition, there is a variance in samples in terms of resolution and image positioning, and the dataset images contain images for one knee.

In contrast, the other images contain images of two knees. So, we augment the training and validation sets, but the testing set is used without any augmentation to test the proposed model accurately. Because any augmentation to the test set will lead to high measures, but it will not be accurate and doesn’t give a sense of reliability.

**Fig. 16:**
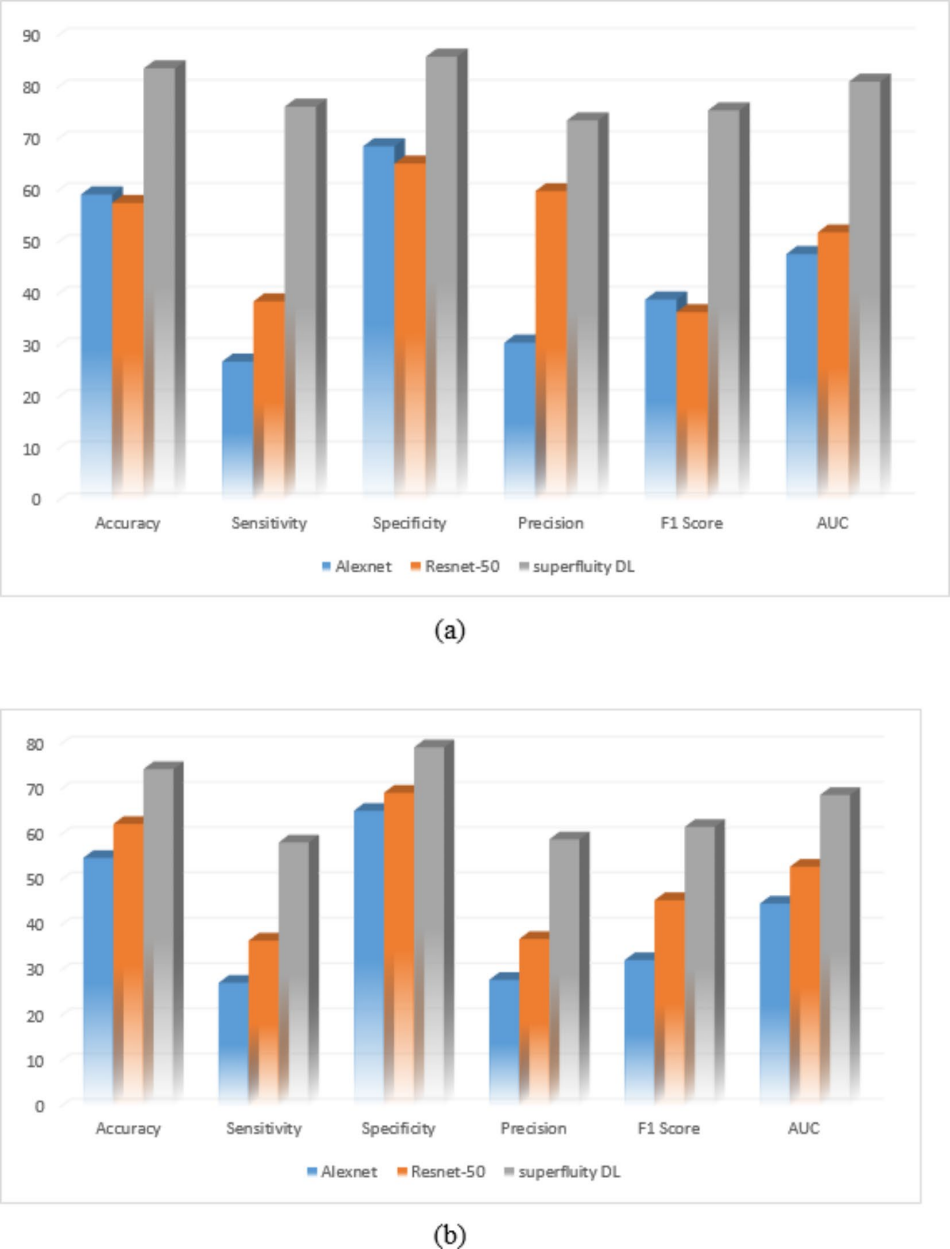
(a) Dataset 1 comparison after augmentation, (b) Dataset 2 comparison after augmentation.

**Fig. 17:**
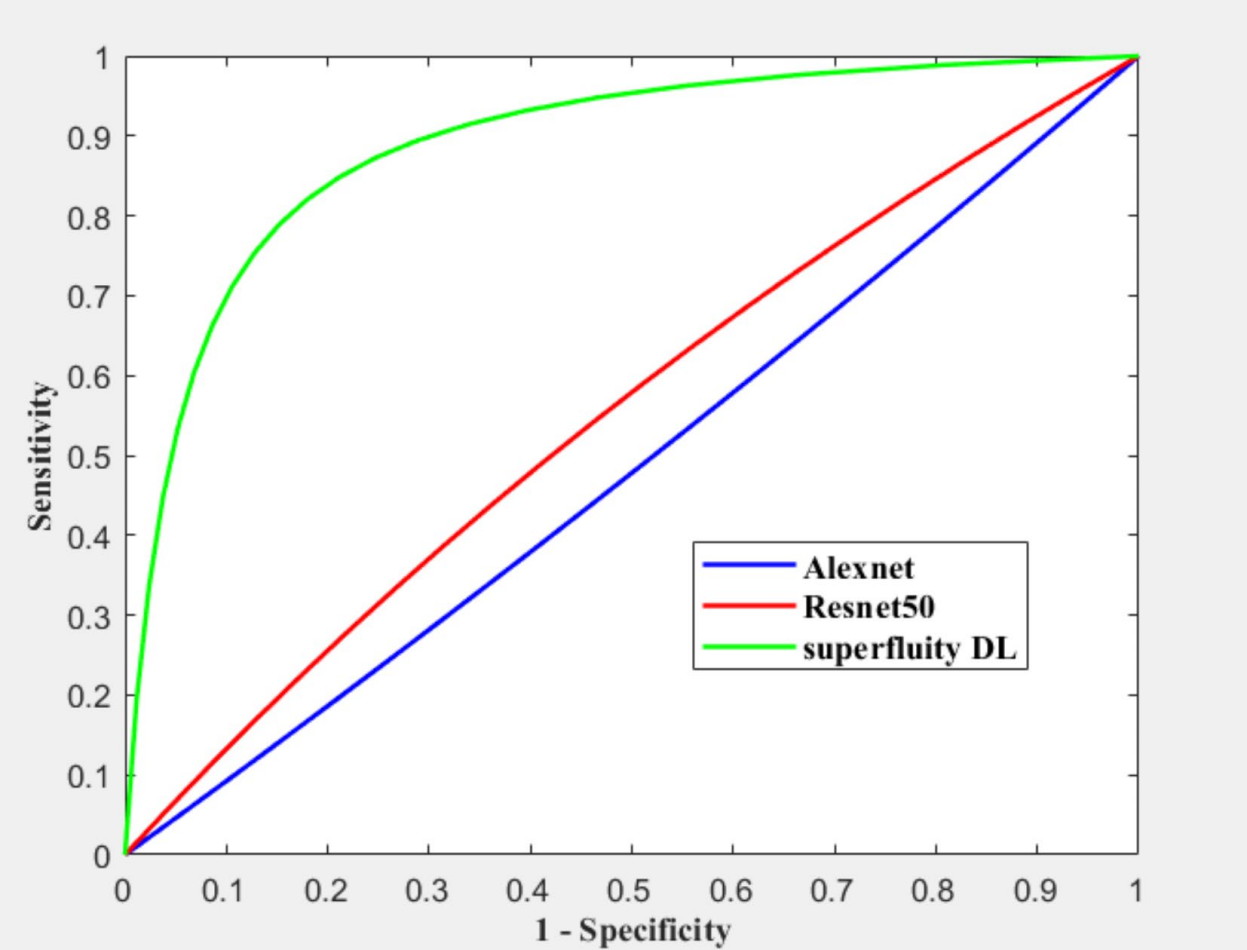
ROC for dataset 1 after augmentation.

**Fig. 18:**
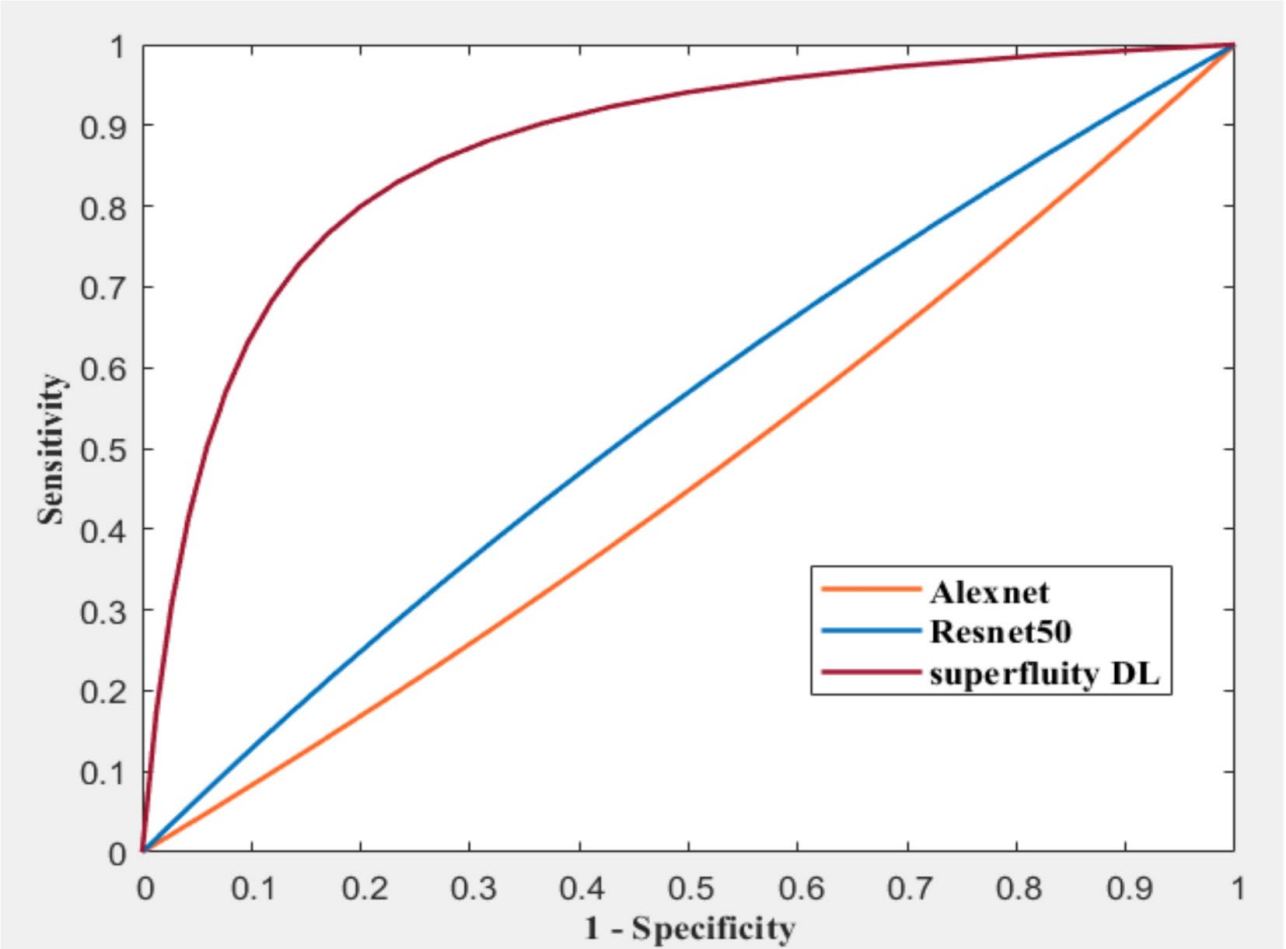
ROC for dataset 2 after augmentation.

Figure 16; Tables 4 and 5 summarize the results obtained after augmentation. The ROC for Datasets 1 and 2 are shown in Figs. 17 and 18, respectively.

**Table 4:** Performance measures for dataset 1 after augmentation.

	Accuracy (%)	Sensitivity (%)	Specificity (%)	Precision (%)	F1 Score (%)	AUC (%)
Alexnet	59.35	27	68.67	30.67	39.02	47.84
Resnet-50	57.72	38.67	65.33	60	36.59	52
Superfluity DL	**83.74**	**76.33**	**86**	**73.67**	**75.61**	**81.16**

**Table 5:** Performance measures for dataset 2 after augmentation.

	Accuracy (%)	Sensitivity (%)	Specificity (%)	Precision (%)	F1 Score (%)	AUC (%)
Alexnet	54.92	27.33	65.33	28	32.35	44.83
Resnet-50	62.42	36.67	69.3	37	45.59	52.99
Superfluity DL	**74.51**	**58.33**	**79.33**	**59**	**61.76**	**68.83**

The previous results show that the pre-trained model fails to classify the knee as normal, osteoporosis, or osteopenia even after augmentation. Alexnet obtained lower results after augmentation than using the original dataset images without augmentation using dataset 2. In contrast, the proposed superfluity DL model achieved higher results than the pre-trained model using datasets 1 and 2.

According to our knowledge, this is the first study to classify normal, osteopenia, and osteoporosis in the knee utilizing deep learning and superfluity mechanisms performed on conventional X-rays. The findings showed that the DL method might have the ability to automatically classify normal, osteopenia, and osteoporosis in knees in the actual world. The proposed model’s accuracy increased by increasing the image samples through different augmentation methods for previous results. Therefore, increasing the image sample led to an increase in the accuracy of the DL model and the accurate classification of the images. Two different datasets have been used to test the ability of the proposed method to classify three forms of the knee into normal, Osteoporosis, and Osteopenia.

Comparison with state of the art is an essential step to prove the ability of the proposed method against others. We utilized two benchmark datasets. To our best knowledge, no literature was delivered using the same datasets utilized in this research except only one study was developed to detect osteoporosis from knee X-rays by Wani and Arora^16^. They utilized 4 well-known models such as AlexNet, vgg19, vgg16, and ResNet. They trained these models from scratch and utilized them as a pre-trained model. The best accuracy was 78.95% achieved by Alexnet. Comparing the obtained results with the proposed method we found that the proposed method beat the performance measure from Wani and Arora^15^. Kumar et al.^17^ proposed a deep-learning model to classify Osteoporosis utilizing X-ray images. They achieved an accuracy rate of 82.61%. Xie et al.^18^ finetuned a deep convolutional neural network knee x-rays. Table 6 compares the obtained results from the proposed method against state of the art.

**Table 6:** Comparison with state of the art.

	Normal / pre-trained CNN	Accuracy	Sensitivity	Specificity	Precision	F1 Score	AUC
Xie et al. ^18^	Pre-trained	73	75	58.2	71	72	66.6
Wani and Arora ^15^	Normal	78.95	-	-	-	-	-
Kumar et al. ^17^	-	82.61	-	-	-	-	-
Proposed method	Normal	**83.74**	**76.33**	**86**	**73.67**	**75.61**	**81.16**

Finally, there are some limitations. The generalizability of our findings is indeed a significant consideration. While our sample size was discussed previously for each dataset separately, we acknowledge that it may not fully represent the entire target population. Additionally, the data we used benchmark datasets which may introduce potential biases. Regarding data quality, the quality of datasets is not standardized which means the accuracy of the proposed method is not the same each time er randomly separate the dataset for training, validating, and testing there is always a risk of measurement error or missing data, which could affect the overall validity of our results.

## Ablation study

Strengthening and improving the model’s groundwork will make it more accurate and dependable overall, which is the aim of the ablation study. In CNN-based applications, an ablation study is frequently performed to evaluate the model’s performance and robustness by removing or modifying different layers and hyper-parameters. Depending on how the design of these components is changed, the performance of this network concerning the main model may rise, fall, or stay the same. On the other hand, an ablation study can be run by comparing the performance of the proposed method against literature that proposed a deep learning model containing more or lower number of layers. As we discussed before we utilized two different pre-trained models Alexnet and Resnet50. These models contain a smaller number of layers than the proposed method. By comparing the obtained results of the two pre-trained models and the proposed model we noticed that the proposed method obtained higher measures than Alexnet and Resnet50. Finally, we evaluated how adding and removing layers affected the accuracy of the classification rate. As discussed previously, the ablations prove that the proposed method was the best measure against adding or removing layers as mentioned in the literature, and using pre-trained models.

## Conclusion and future work

In this study, we proposed a novel DL model using a superfluity mechanism to classify the knee X-ray into normal, osteopenia, or osteoporosis. The proposed model uses superfluity learning to overcome image degradation and scarcity by enhancing information flow and reformulating layers. It passes extracted features through two paths, concatenating them together in a superfluity block, which adds input directly to the output. We proposed two superfluity blocks in addition different filter sizes are used to generalize the model and to help extract features accurately. According to our knowledge, there is no published work on the same dataset after being labeled. We apply transfer learning to the pre-trained DL architectures Alexnet and Resnet-50 to compare the proposed DL model. To generalize the proposed model, we trained, validated, and tested the proposed model on two datasets. The findings showed that the proposed superfluity deep learning model performed better than pre-trained models. This advantage is particularly evident when dealing with small or imbalanced datasets. Superfluity has the potential to accelerate convergence and stabilize the training process. The proposed Superfluity DL achieves the best accuracy compared to the other pre-trained models.

Conversely, including superfluity often leads to increased model complexity, making the model harder to interpret, train, and deploy, especially in resource-constrained environments. Superfluity models may require more computational resources for training and inference due to increased parameters and operations, which limit their practicality in certain applications. Training such a model might be challenging and finding the optimal parameters can be more complex than simpler architectures, which may require more advanced optimization techniques. If not carefully designed and implemented, superfluity may introduce the risk of model degradation. The effectiveness of superfluity can depend on the training data’s quality and diversity. If the data is not representative, the model may not fully exploit the benefits of the redundant pathways.

## Data Availability

Data is available on request from the corresponding author (Khalid M. Hosny).

## Abbreviations

DL: Deep Learning
BMD: Bone mass density
DEXA: Dual-energy X-ray absorptiometry
AI: Artificial Intelligence
CNN: Convolution neural networks
CAD: Computer-aided diagnosis systems
ROC: Receiver operating characteristic
